# Cryogenic Drilling of AZ31 Magnesium Syntactic Foams

**DOI:** 10.3390/ma13184094

**Published:** 2020-09-15

**Authors:** Sathish Kannan, Salman Pervaiz, Muhammad Pervej Jahan, DoraiSwamy Venkatraghaven

**Affiliations:** 1 Department of Mechanical Engineering, American University of Sharjah, Sharjah 26666, UAE; skannan@aus.edu; 2 Department of Mechanical and Industrial Engineering, Rochester Institute of Technology—Dubai Campus, Dubai 341055, UAE; 3 Department of Mechanical and Manufacturing Engineering, Miami University, Oxford, OH 45056, USA; jahanmp@miamiOH.edu; 4 SWAMEQUIP Ltd., Chennai 600064, India; swamequipchennai@gmail.com

**Keywords:** drilling, cryogenic cooling, surface roughness, AZ31, syntactic foam

## Abstract

Machined surface quality and integrity affect the corrosion performance of AZ31 magnesium composites. These novel materials are preferred for temporary orthopedic and vascular implants. In this paper, the drilling performance of AZ31-magnesium reinforced with hollow alumina microsphere syntactic foam under LN2 cryogenic, dry, and Almag^®^ Oil is presented. Cutting tests were conducted using TiAlN physical vapor deposition (PVD) coated multilayer carbide and K10 uncoated carbide twist drills. AZ31 magnesium matrices were reinforced with hollow alumina ceramic microspheres with varying volume fractions (5%, 10%, 15%) and average bubble sizes. Experimental results showed that the drilling thrust forces increased by 250% with increasing feed rate (0.05 to 0.6 mm/tooth) and 46% with the increasing volume fraction of alumina microspheres (5% to 15%). Cryogenic machining generated 45% higher thrust forces compared to dry and wet machining. The higher the volume fraction and the finer the average size of hollow microspheres, the higher were the thrust forces. Cryogenic machining (0.42 µm) produced a 75% improvement in surface roughness (Ra) values compared to wet machining (1.84 µm) with minimal subsurface machining-induced defects. Surface quality deteriorated by 129% with an increasing volume fraction of alumina microspheres (0.61 µm to 1.4 µm). Burr height reduction of 53% was achieved with cryogenic machining (60 µm) compared to dry machining (130 µm). Overall, compared to dry and wet machining methods, cryogenic drilling can be employed for the machining of AZ31 magnesium syntactic foams to achieve good surface quality and integrity.

## 1. Introduction

Magnesium being the lightest metal, is commercially available with enhanced mechanical properties [1]. Magnesium-based closed cell syntactic foams possess great potential in important sectors such as biomedical, automotive, and marine applications to design lightweight products [2]. Due to their excellent biocompatibility, magnesium-based alloys are potential candidates for temporary biodegradable medical implants [3,4,5]. The corrosion behavior of magnesium-based alloys is controlled through the modification of their surface characteristics through enhanced coating compositions, the choice of machining methods, and various mechanical working methods [6]. It has been shown that an increase in the volume fraction of hollow microspheres increases the hardness, compression yield strength, peak compression strength, and plateau strength of the syntactic metal foams [7,8,9,10]. The coarser the average size of hollow reinforcements is, the lower the hardness of the magnesium closed-cell foam [11,12]. The mechanical properties of aluminum-based syntactic foam are shown to be comparable to the base alloy under cryogenic conditions [8].

Cryogenic machining, which is carried out as a secondary operation to produce features such as holes, internal threads, etc. is shown to alter grain size, machined subsurface residual stresses, and crystallographic texture [13,14]. These studies have shown that an appropriate selection of machining methods can be used to control the corrosion rate of magnesium alloys for the manufacture of engineering components. Cutting tools with large edge radii along with the application of cryogenic coolant has been shown to enhance the surface integrity of AZ31 magnesium alloy [14]. Constitutive modelling of an AZ31B-O magnesium alloy was carried out by Giraud et al. [15]. They reported that the stress was not sensitive to applied strain rate at temperature below 200 °C. However, when the temperature increased above 200 °C, the stress was found to be sensitive of strain rate and the material softening due to dynamic recrystallization was observed. The effect of process parameters, cutting tool rake angle, and tool edge radius on cutting mechanics and surface integrity during cutting AZ31B was studied under different cooling conditions by Outeiro et al. [6]. It is reported that higher undeformed chip thickness values required minimum specific cutting energy. Machining using the cryogenic cooling method resulted in higher subsurface compressive residual stress with reduced thickness of the deformed layer [8]. Ugur et al. [16], performed drilling tests on AZ31 magnesium alloy under dry and cryogenic conditions. The results of tests from dipped cryogenic drilling showed lower tool wear, smaller chips, and reduced adhesion of chips. However, the application of cryogenic cooling resulted in higher thrust force compared to dry machining. No spark or ignition of chips was reported during dipped cryogenic drilling. Beatriz et al. [17] studied the influence of drilling conditions on average surface roughness achieved during machining AZ91D-F magnesium alloy. Results of their optimization showed a good quality surface could be achieved at a feed rate of 0.05 mm/rev and 40 m/min cutting speed with a drill point angle of 135°. In another study, Brian et al. [18] studied chip morphology and chip formation mechanisms during cutting magnesium metal matrix composite reinforced with solid micro- and nanoparticles of silicon carbide. In their study, the cutting conditions were varied between 0.1 to 10.0 m/s along with tool rake angle, volume fraction, and the average size of silicon carbide microparticles. Through their study under dry cutting conditions, it is shown that the chip morphology changes from saw-tooth to more discontinuous particle type powder with increasing cutting speed. The introduction of ceramic reinforcements increases the brittleness of the matrix [8]. Chip morphology is reported to be more dominant on cutting speed compared to tool rake angle, volume fraction, and the average size of silicon carbide [18]. Mustafa et al. [19] conducted drilling studies on graphene nanoplatelet, and silicon carbide reinforced in a hybrid magnesium matrix composite, which was manufactured through the powder metallurgy method. The results of their study showed that lower thrust forces could be achieved using the physical vapor deposition (PVD) coated tool and drilling at a cutting speed of 50 m/min and a feed rate of 0.10 mm/rev. Their study shows that lower feed rates are preferred while drilling hybrid magnesium metal matrix composites primarily due to the achievement of good surface finish and lower thrust forces [19]. Dry microdrilling tests were carried out on pure magnesium reinforced with the varying volume fraction of nanoparticles of SiO_2_ [20]. Chip morphology was found to change from short spiral type to powder type with the increasing volume fraction of SiO_2_. Surface roughness was found to deteriorate with an increase in volume fraction and at higher feed rate. It is shown that by reducing feed rate and rotational speed, the height of burrs could be reduced [20,21]. Cryogenic drilling of AZ31B magnesium alloy has been studied through finite element modeling. The study showed an increase in machined surface hardness compared to dry machining [22,23]. The influence of the cooling method during drilling AM60 magnesium alloy has been studied experimentally. It has been reported that the minimum quantity lubrication method produced more uniform thrust force and discontinuous chips. Hole surfaces are also shown to be of better surface finish [24]. The development of an appropriate cooling method for machining magnesium-based composite foams is very important from the perspective of mass production. Sustainable cooling methods such as dry machining, minimum quantity lubrication, and cryogenic machining are key technologies worth investigating.

As the literature review shows that in the field of cryogenic machining, machinability studies on drilling of magnesium metal matrix syntactic foams are very limited. Not many studies have been carried out to study the machined surface quality and integrity of AZ31 magnesium matrix syntactic foams and hence the focus of this research. Syntactic foam materials are classified as difficult-to-cut due to its heterogeneous microstructure and inherent brittleness of the reinforcing hollow ceramic microsphere phase [11]. Machining of magnesium-based syntactic foam presents significant challenges in the form of dimensional accuracy due to edge delamination and disintegration. Besides, the formation of built-up-edge, machining-induced defects primarily due to fracture of hollow ceramic microsphere poses severe surface integrity issues. Subsequently, advanced manufacturing strategies, that is, optimized cutting parameters, cooling lubrication methods, etc. are mandatory for efficient drilling of AZ31 magnesium syntactic foams. Some of the key performance indicators such as cutting force, surface quality and integrity, and burr formation has been covered in this study with special attention on hole drilling operation.

## 2. Material and Methods

Magnesium syntactic foam billets were manufactured through the squeeze casting method [11]. During the casting process, the stirrer RPM was maintained between 450–500 RPM for 10 min. The temperature of the melt was 750 °C under inert ultrahigh purity argon gas (3 L per minute), and the squeeze pressure was 117 MPa. The size of the castings was 50 mm diameter and 200 mm long. The electromagnetic vibrator was used at 300 Hz to disperse the reinforcements into the melt. The mold was preheated to 300 °C, and the hollow microspheres were preheated to 200 °C. The chemical composition of hollow aluminum oxide (Table 1) was obtained from the supplier (Pacific Rundum Co., Ltd., Tokyo, Japan).

### 2.1. Cutting Tool

**Figure 1:**
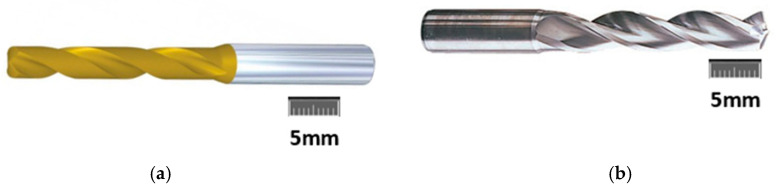
Carbide twist drills: (**a**) TiAlN multilayer fine-grain grade KC7325; (**b**) uncoated carbide—K10.

Kennametal TM φ5 mm carbide twist drills comprising of a multilayer TiAlN-PVD-coated universal fine-grain grade and a K10 uncoated twist drill were used in this study (Figure 1). The TiAlN-PVD-coated drill has superior properties of wear resistance for machining abrasive composite materials and are more efficient than TiN-PVD grades. Twist drill properties are shown in Table 2. New drills were used for each cutting test.

### 2.2. Cutting Conditions

Drilling test conditions used in this study are shown in Table 3. Hole-drilling experiments were conducted over a range of cutting speed and feed/tooth conditions on billets reinforced with varying volume fraction and average microbubble AZ31 syntactic foams. Tests were carried out under dry, wet, and cryogenic cooling conditions on a Doosan DNM-4500 CNC machine (Seoul, South Korea). The cutting conditions were determined based on preliminary test results. Holes of 5 mm depth were drilled on the workpiece material. Three holes have been drilled under each state. Tool wear morphology was observed under a scanning electron microscope. A distance of 10 mm was provided to avoid the work hardening around the drilled holes.

### 2.3. Cooling Conditions

**Figure 2:**
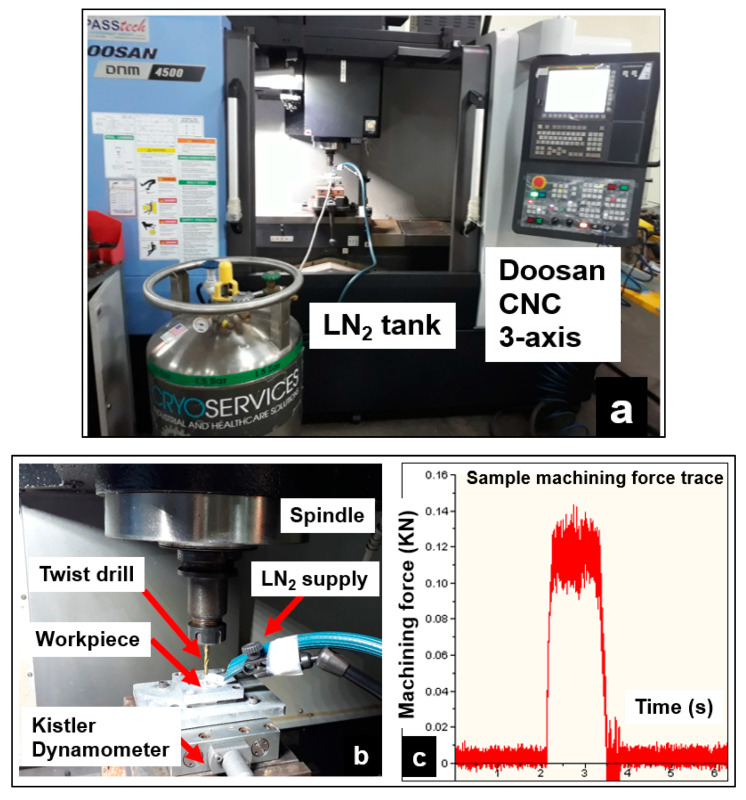
(**a**) Experiment set up; (**b**) Cutting force dynamometer; (**c**) Cutting force signal.

The operational performance of AZ31 syntactic foams, particularly in biomedical and automotive applications, demands excellent surface quality and integrity requirements. This necessitates the need to down select the appropriate cooling conditions to achieve this requirement. Dry cutting is considered a green manufacturing process that helps to eliminate environmental pollution and reduce manufacturing costs. In this study, dry machining, along with the aid of compressed air to remove the dry chips, has been employed. Compressed air helps to clear the loose chips from the cutting zone and prevent hazardous chip ignition, which is common during magnesium machining. Wet cutting conducted in this study used Almag^®^ Oil, which is a transparent, low-viscosity, straight mineral cutting oil. This cutting oil is designed mainly for use in light-duty machining operations on magnesium and their alloys. It contains no water to react with magnesium to form heat and explosive hydrogen. For the cryogenic cooling method, liquid nitrogen (LN_2_) was sprayed at a constant pressure of 2 bar. The cryogenic experimental set up is shown in Figure 2. A KISTLER™ 9129AA three-channel dynamometer was used along with a multichannel charge amplifier type 5080 to measure the machining forces.

### 2.4. Surface Roughness, Burr, and Chip Morphology

Surface quality and integrity of drilled syntactic foams are of utmost importance for their in-service performance. The drilled hole surface quality and integrity were assessed using a Zeiss confocal microscope. The holes were sectioned parallel to their axis, and surface roughness measurements were made at different locations in the hole. The mean values of average surface roughness (Ra) were noted. To assess the machining induced defects at the entry, bore section, and exit side of the hole, the drilled sample surfaces were analyzed using a scanning electron microscope. The drilling process created burrs at the entry and exit side of the hole, which was measured using a Zeiss microscope and assessed using a scanning electron microscope. Burr height was measured at both entry and exit sides of the hole at different locations, and the average value has been reported. The chips were collected after every test for analysis of their form.

## 3. Results and Discussion

### 3.1. Thrust Forces

**Figure 3:**
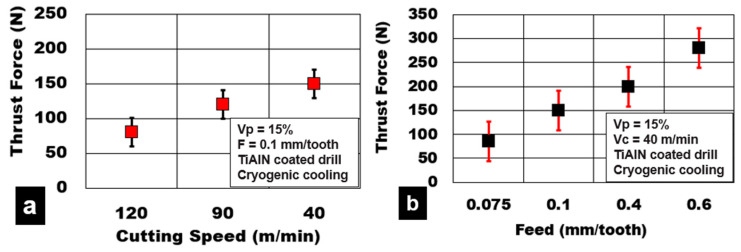
Effect of process parameters during plunge drilling of AZ31-alumina syntactic foam (**a**). effect of cutting speed; (**b**). effect of feed.

The effect of process parameters on test results shows that the variation in cutting speed and feed/tooth has an important influence on the magnitude of generated thrust force. The average thrust force values during cutting AZ31-15% hollow alumina microbubbles under different drilling conditions are shown in Figure 3. An increase in cutting speed from 40 m/min to 120 m/min decreases the thrust force by 46% (150 N to 80 N). This is primarily attributed to the softening of the AZ31 matrix due to an increase in shear zone heat generation. A reduction of the contact area at the cutting zone and the reduction of the specific cutting energy is also expected. On the other hand, an increase in feed/tooth value from 0.075 mm/tooth to 0.6 mm/tooth and an increase in thrust force by 250% is recorded (85 N to 300 N). Higher feed/tooth numbers cause an increase in chip load and volume of metal removed, leading to an increase in chip tool contact length. Adhesion of magnesium matrix and abrasion of the hollow ceramic microsphere on the tool are the main causes of tool wear during drilling. High friction causes the generation of higher thrust forces at higher feed rates.

**Figure 4:**
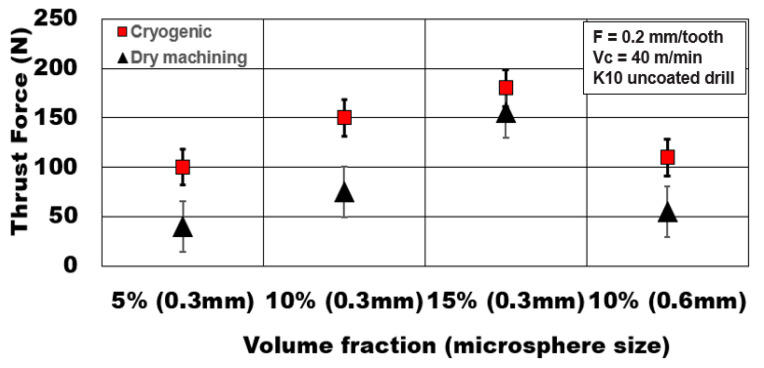
Effect of hollow microsphere volume fraction and average size on thrust force during plunge drilling of AZ31-alumina syntactic foam.

Figure 4 shows the influence of the volume fraction of hollow alumina microbubbles reinforced in the AZ31 matrix on the magnitude of thrust forces generated. An increase in the number of microspheres engaged during metal cutting results at higher volume fractions. Thrust forces increase by 80% as volume fraction increases from 5% to 15% under cryogenic cooling. However, under dry machining, the increase in thrust force is almost 287% with increasing volume fraction. This shows the consistency of thrust forces under cryogenic machining. The presence of microbubbles enhances the compressive yield strength and peak strength of the syntactic foam [8,12]. This phenomenon results in higher thrust forces.

On the other hand, as the average alumina microsphere size becomes coarser, the number of bubbles in the matrix reduces. The finer the bubbles are, the higher the peak compressive strength of the metallic foam [12]. This phenomenon results in higher thrust forces (almost 1.5 times) generated compared to foams reinforced with coarse microspheres. This result shows the significance of the average microbubble size and their influence on the plastic deformation behavior of the AZ31 Mg matrix during the plunge drilling process.

**Figure 5:**
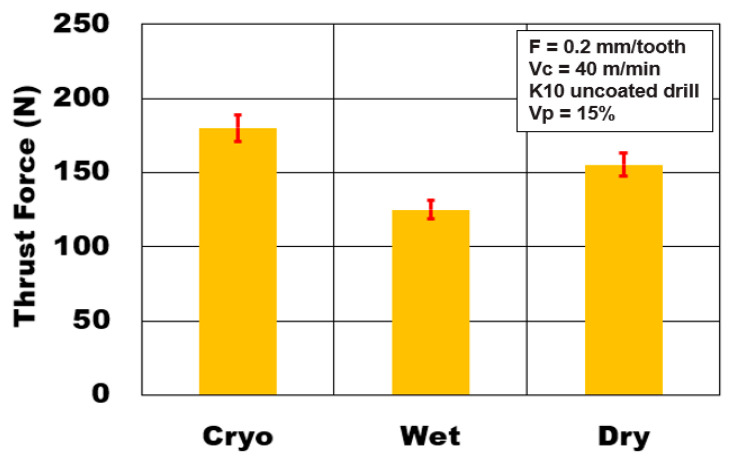
Effect of cooling medium on thrust force during plunge drilling of AZ31-alumina syntactic foam.

The effect of different cooling methods on thrust forces generated during drilling AZ31-15% volume fraction of hollow alumina microbubbles is shown in Figure 5. The experiment results show that the type of cooling/lubrication medium employed during drilling has an important role to play on the magnitude of thrust forces. It is seen that cryogenic cooling (180 N) increases the thrust forces by 45% and 16% compared to wet machining (125 N) and dry machining (155 N) conditions, respectively. Reduction in cooling temperature to subzero conditions causes an increase in strength of the workpiece material and, hence, higher thrust force, and agrees with the reported literature [9,25,26]. Sudden cooling could promote the hardening of the magnesium matrix and enhance its brittleness. This causes preferential propagation of transverse matrix cracks and longitudinal interface cracks leading to rapid debonding of reinforcements. The ceramic alumina microsphere cracking is also affected due to the rapid cooling process [10]. It has been highlighted in the literature that material yield strength and hardness of the closed cell syntactic foam increases as testing temperature decreases [8,9]. Experimental results indicate a transformation in the plastic deformation behavior of the workpiece material with a change in the cooling method in the form of variation in the magnitude of machining forces. Thrust forces generated during dry machining conditions were 25% higher than the wet cooling conditions. The wet cooling method using Almag^®^ Oil recorded the lowest of the thrust forces generated. The use of mineral oil-based coolant helps to minimize the magnesium chip adhesion on to the cutting tool, thus reducing friction and built-up-edge This contributes to a reduction in thrust forces generated.

During dry machining, the effect of heat generation during the drilling process could have resulted in the AZ31 matrix thermal softening and a higher slip of shear planes. This phenomenon initiates the plastic deformation of the soft matrix around the microsphere. This leads to bubble separation and pull out from the matrix. This alters the load transfer mechanism between the alumina microsphere and the matrix. Higher plasticity of the matrix promotes magnesium adhesion in the form of built-up edge and high friction on the cutting tool edge leading to high thrust force. Whereas in the case of cryogenic machining, a high work-hardened matrix carries the load and enables higher peak strength of the material resulting in higher thrust force. This explains the influence of the cooling/lubrication method on the magnitude of thrust force generated during drilling AZ31-15% alumina metallic foam.

### 3.2. Surface Quality and Integrity

**Figure 6:**
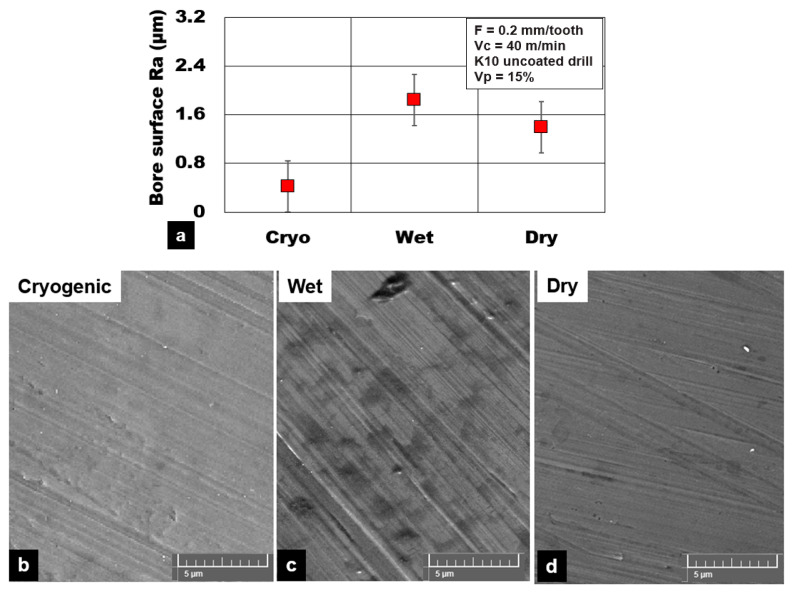
Effect of cooling medium on machined bore surface roughness (Ra) during plunge drilling of AZ31-15% hollow alumina syntactic foam. (**a**) Graphical illustration; (**b**) cryogenic; (**c**) wet; (**d**) dry.

Figure 6 shows the average surface roughness (Ra) values under different cooling conditions during plunge drilling of AZ31 magnesium reinforced with a 15% volume fraction of hollow alumina ceramics. The lowest values of surface roughness Ra were recorded under cryogenic cooling conditions (0.42 µm) in comparison to dry machining (1.4 µm) and wet cooling (1.84 µm). This is an almost 75% improvement in surface quality with cryogenic machining. Although higher thrust forces were recorded with cryogenic machining, it produces consistently good bore surfaces with less machining induced damages. Cryogenic cooling promotes a favorable condition of brittle fracture of chips that helps to maintain a good surface finish with reduced chip smearing, smaller feed marks, and reduced plasticity of the magnesium matrix (Figure 6). During dry and wet machining, the extent of plastic deformation and material side flow of the AZ31 matrix around the cutting edge was more prominent than cryogenic cutting. This plastic flow of the AZ31 matrix around the hollow microspheres results in an increased width and depth of feed marks (Figure 6). The higher cutting temperature during dry machining results in adhesion of chips, causing deterioration of the machined bore surface.

**Figure 7:**
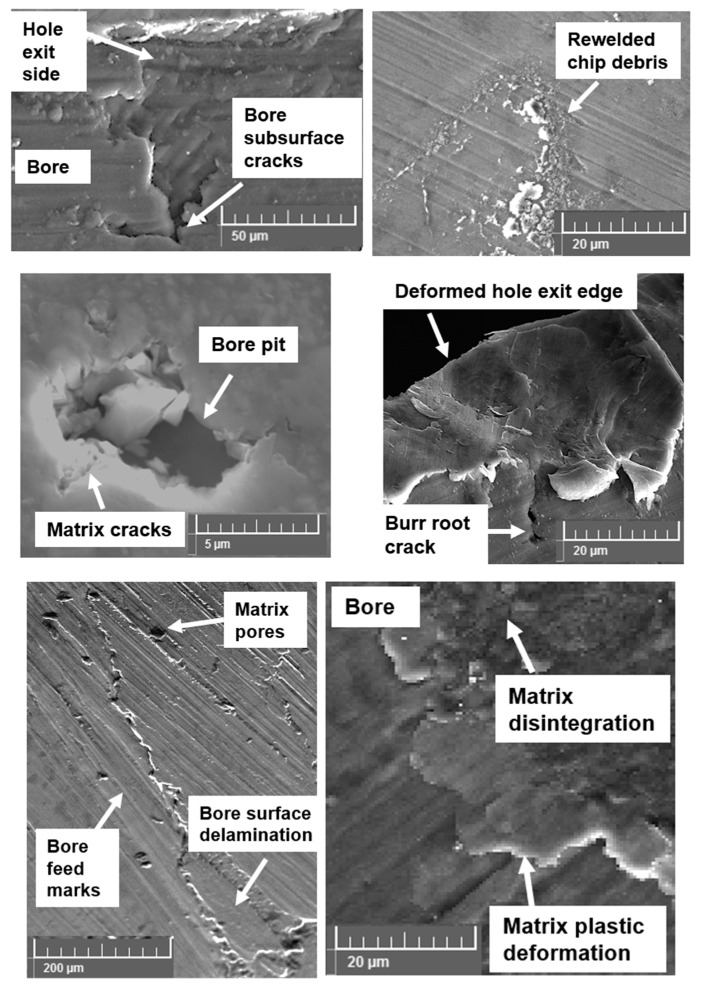
Machining induced defects in the hole bore section during plunge drilling of AZ31-alumina syntactic foam.

Several machining induced defects were noticed during drilling AZ31 magnesium syntactic foams. In the case of dry machining, the primary surface defects were found to be material smearing and dry chips rewelded back on to the drilled surface. Despite the application of compressed air during dry machining, fine chips were rewelded on to the machined surface, especially towards the exit side of the drilled hole (Figure 7). Along with this, other key surface integrity issues recorded with dry machining were primarily AZ31 magnesium matrix plastic deformation, disintegration, and material side flow. Although wet machining provided the much-required cooling and lubrication effect, the required surface finish was not achieved with this type of cutting method. Wet machining produced surfaces with a higher percentage of bore pits, and more feed marks were observed in the machined surface. Bore voids and pits were primarily due to pulling out of the hollow reinforcement from the matrix due to the flushing of the loose microspheres by the cutting fluid. In the case of the cryogenic cooling method, key surface anomalies were primarily matrix delamination, disintegration, and subsurface crack propagation from the exit edge of the hole. The initiation of these defects during cryogenic machining is mainly due to brittle fracture of the material. This damage phenomenon affects the drilled hole edge definition.

**Figure 8:**
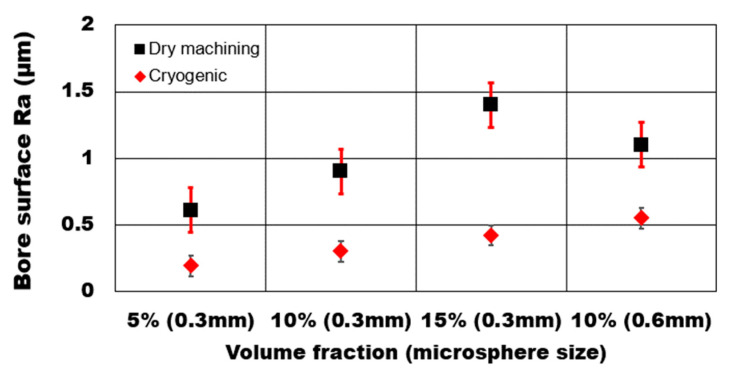
Effect of volume fraction and microsphere size on machined bore surface roughness (Ra) during plunge drilling of AZ31-alumina syntactic foam.

The effect of hollow microsphere volume fraction and average bubble size on achievable average surface roughness Ra during dry drilling of AZ31 magnesium syntactic foam is shown in Figure 8. The presence of microspheres greatly influences the extent of machining induced surface defects. An increase in the volume fraction of the microsphere increases the number of hollow ceramic particles in the AZ31 matrix. As a result, the number of defect generation sites increase in the matrix. As can be seen from the results, the average surface roughness (Ra) values increased drastically by 129% with the increasing volume fraction of hollow alumina reinforcements from 5% to 15%. The defects recorded during the SEM analysis of the machined surface showed a higher percentage of microsphere damage sites that resulted in microsphere cracking and pulled out in higher volume fraction syntactic foams. Consequently, surface pits and voids were induced in the drilled bore and the hole entry and exit edges.

During dry machining, syntactic foam reinforced with a lower percentage of microspheres showed higher plastically, and material side flow compared to higher volume fraction foams. Softer AZ31 matrix undergoes plastic deformation once the yield point is reached before microsphere fracture. This type of deformation mechanism results in the debonding of the microspheres from the matrix. The effect of the average microsphere size on the average surface roughness was studied. It is noted that, during dry machining, an increase in hollow reinforcement average size (Ra: 1.1 µm) results in a surface that is 22% rougher than the finer hollow reinforcements (Ra: 0.9 µm). The percentage increase in surface roughness for foam with a coarser microsphere (0.55 µm) is higher during cryogenic machining at 83% than foam with finer microspheres (0.3 µm). The presence of a fine microsphere in the magnesium matrix enhances the hardening behavior of the matrix with more uniform shear deformation with controlled chip formation. Debonding and pull-out of a coarse microsphere from the matrix leaves a large crater and higher densification of the matrix. Promotion in the plastic movement of the matrix during the densification process causes a rough surface in the case of coarse microsphere foams.

### 3.3. Burr Formation

**Figure 9:**
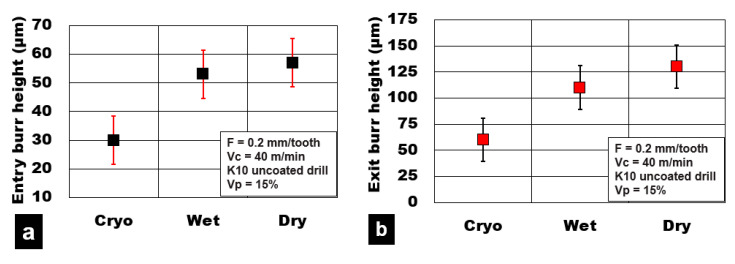
Effect of cooling medium on hole burr height during plunge drilling of AZ31-alumina syntactic foam. (**a**) Entry burr height; (**b**) Exit burr height.

Figure 9 shows the burr height values measured at the hole entry and exit locations on the holes, respectively. The majority of the dry machining conditions produced burrs due to material push-out induced due to thermomechanical loads that act during the drilling process. It is well known that during dry machining conditions, the AZ31 magnesium plasticity is enhanced due to high heat generation in the shear zone. This increases the cutting temperature and torque value required to shear through the material compared to cryogenic and wet machining conditions. Observation of the machined surface shows that dry cutting produced higher plastic deformation of the AZ31 magnesium matrix and material side flow, resulting in higher burr formation. On the other hand, cryogenic cutting conditions (60 µm) resulted in at least 55% lower values of burr height compared to wet machining (115 µm) and dry machining (130 µm). Compared with exit hole edges, burr heights at the entry side of the holes on an average were 45% lower for all cutting conditions considered in this study. Wet machining produced edges that had 15% lower values of burr height than dry machining conditions.

**Figure 10:**
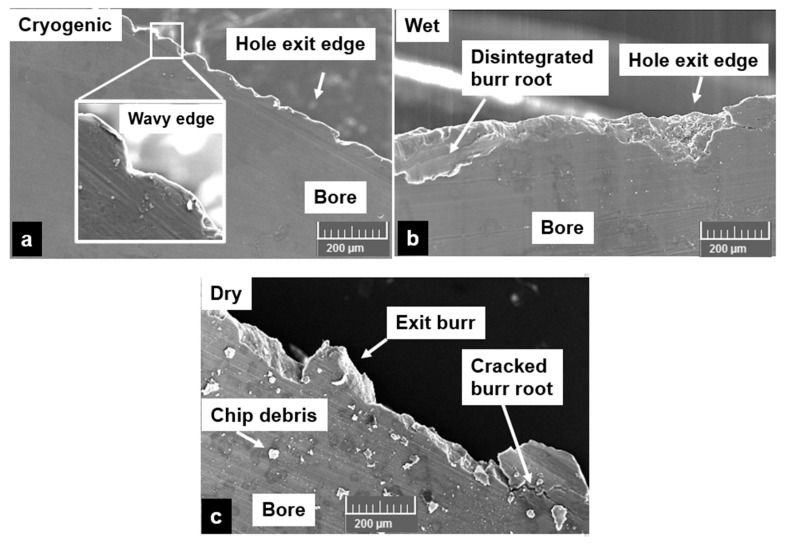
Burr types at the exit side of the hole: (**a**) cryogenic, (**b**) wet, (**c**) dry.

The types of burr formed at the exit holes were analyzed using an SEM. It was observed from the SEM micrographs that the hole edge forms produced by cryogenic machining were wavy (Figure 10a). This shows that the cryogenic machining has resulted in a reduction of the ductility of the AZ31 magnesium matrix causing brittle failure of the machined edges. SEM images also show that pressure coolant is not favorable for cutting syntactic foams due to enhanced brittleness of the material. This could lead to instant cracking of burr root and take along a portion of the hole edge leading to loss of hole roundness due to edge delamination and disintegration (Figure 10b). Several such locations on the exit hole edges were observed during wet cutting conditions. On the other hand, dry machining showed a higher tendency to form a combination of Poisson and roll-over burrs. This is primarily due to higher ductility of the AZ31 matrix causing enhanced plastic deformation and material push out. In summary, AZ31-15 vol% syntactic foam produces a burr that is brittle and has the tendency to disintegrate due to preferential propagation of the burr root crack under cryogenic and wet cooling. This mechanism can significantly affect the subsurface integrity of the machined hole.

**Figure 11:**
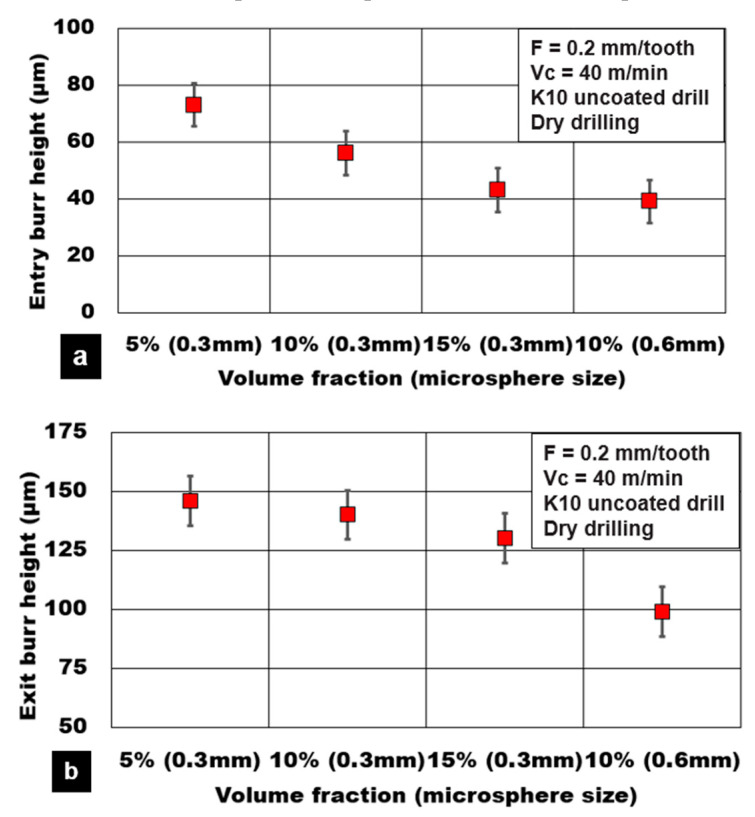
Effect of hollow microsphere volume fraction and average size on hole burr height during plunge drilling of AZ31-alumina syntactic foam. (**a**) Entry burr height; (**b**) Exit burr height.

The effect of hollow alumina reinforcement on the burr height values is shown in Figure 11. It is noted that the burr height values at the entry and exit side of the hole reduce by 40% and 15%, respectively, with the increasing volume fraction of microspheres. This result points to the increasing brittle fracture of the material for higher volume fraction foam. As the volume fraction increases to 15%, the number of ceramic microspheres in the matrix increases. The constraint in the plastic deformation of the AZ31 matrix is due to pinning by alumina microspheres. As a result, the material side flow is reduced with lower material push out at the exit side of the hole. Pronounced edge chipping and delamination are noticed during cutting of higher-volume fractions. The holes drilled in magnesium syntactic foams reinforced with coarser microspheres resulted in lower burr height values. With an increase in the average microsphere size, the percentage of alumina surface defects in the form of microporosity increases. Compression test results show a reduction in strain to fracture for syntactic foams reinforced with a coarser microsphere [12]. As a result, the brittle material fracture is more pronounced in the case of coarse hollow microsphere reinforced magnesium syntactic foams. The entry and exit burr height values reduced by approximately 70% with AZ31-10% volume fraction foam reinforced with a coarser microsphere compared to finer microspheres.

### 3.4. Chip Morphology

**Figure 12:**
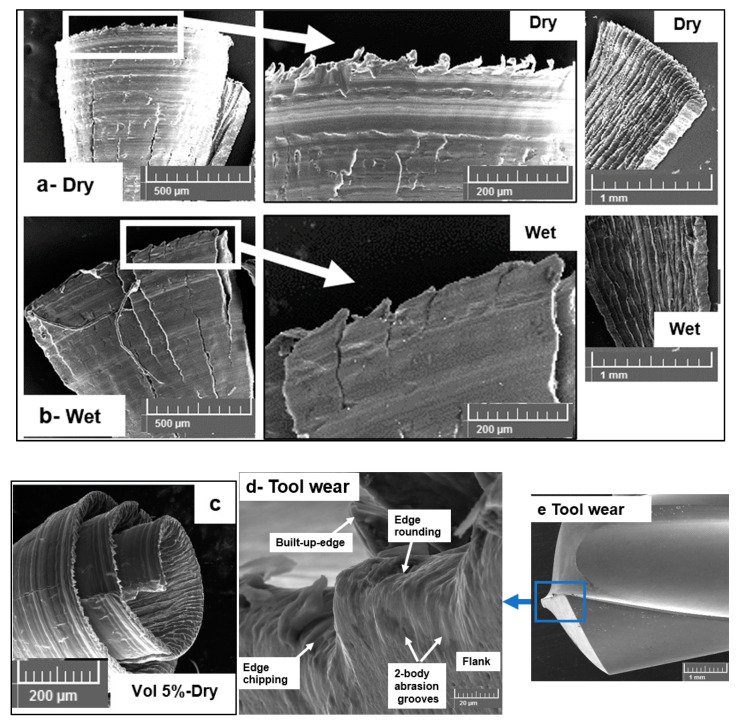
Chip forms during machining AZ31-15% alumina syntactic foam: (**a**) dry cutting, (**b**) wet cutting, (**c**) dry cutting AZ31-5% alumina, (**d**,**e**) tool-wear during cryocooling.

The chip morphologies during the dry and wet machining of the alumina microsphere reinforced AZ31-15% syntactic foams are shown in Figure 12. The method of cooling and lubrication is found to have the most significant effect on the chip morphology. As can be seen from the SEM micrographs, dry machining produced semicontinuous-type chips with minimal edge serrations. Significant plastic deformation on the tool surface of the chip is noticeable on the micrograph with considerable material side flow. This is predominantly due to high friction and heat generation in the deformation zone caused as a result of severe adhesion of AZ31 magnesium with the tool. The length and spacing of the serrations are very small, and uniform as the ductile nature of the formed chip is seen with crack propagation arrested very quickly due to the closing of the ductile magnesium ahead of the crack. On the other hand, wet machining produced chips with a higher number of serrations with considerable deep crack propagation. Significant cracking was observed, which primarily points toward the brittle nature of the formed chip. The chip lamellae serrations were uniformly spaced at approximately 60 µm and 40 µm in crack depth compared to dry machining, which had very small values of less than 10 µm. In general, the chip is formed due to crack initiation at the free surface and propagation towards the tooltip. This mechanism of chip formation agrees well with the reported literature on cutting aluminium- and magnesium-based MMCs [18,27,28]. During the dry cutting of magnesium syntactic foams, key modes of crack initiation have been reported as follows [11]: Coalescence of matrix defects and voids, hollow microsphere debonding and pull-out, and hollow microsphere fracture and collapse followed by matrix densification. The nature of matrix deformation characteristics under dry machining is controlled by its plasticity and flow around the hollow microspheres. The heat generated during the shearing process allows the early yield of the AZ31 matrix. The plastically deforming matrix is subjected to high compressive forces, and load-bearing is transferred through the fractured interface on to the microsphere. The hollow microsphere takes load until its peak strength, at which point it fractures and collapses. Matrix densification follows this step allowing the open voids and microsphere pulled out spaces to be closed and densified. SEM micrographs of the chips collected under dry machining showed extensive plastic deformation, adhesion, and smearing of the material. The extent of plastic deformation, as observed on the free side of the chip through SEM pictures, shows densified material (Figure 12a) closely. This confirms the influence of heat generation and friction during dry machining on the plastic deformation behavior of the AZ31 matrix. Ductile tearing of chips was also observed through SEM pictures.

In the case of wet machining, the heat generation is considerably reduced due to lower friction and built-up-edge formation on the cutting edge. The application of pressure coolant carries out the act of flushing the semifractured or fully fractured microspheres leading to a higher percentage of open voids in the matrix. This helps in the faster coalescence of transverse matrix cracks and interface longitudinal cracks allowing the crack propagation to move along the shear zone. This phenomenon promotes the formation of chips with a higher degree of serration during wet machining. A hardened matrix is also observed through a less densified matrix at the free side of the chip compared to dry machining (Figure 12b). The influence of volume fraction of alumina microspheres on the type of chip morphology is observed using SEM micrographs. Figure 12c shows a typical chip formed while cutting a 5% volume fraction of alumina reinforced in the AZ31 matrix. Through compression tests conducted on syntactic foams, it is clear that an increase in the volume fraction of ceramic microspheres leads to a reduction in material ductility and strain to fracture [7,8]. At a lower volume fraction of microsphere reinforcements (5%), the chips formed were found to be semicontinuous and curly in form compared to discontinuous serrated chips formed during cutting higher volume fraction syntactic foams (15%). A lower volume fraction means less number of alumina microspheres. This shows the ductile nature of the material machined with fewer cracks and defects. A higher degree of plastic deformation of the AZ31 matrix with a highly densified matrix is noticed in the chips of lower volume fraction magnesium syntactic foams. Representative tool wear during drilling AZ31 magnesium-15% alumina syntactic foam is shown in Figure 12d under cryogenic cooling. The flank surface of the drill consists of several two-body grooves that have been caused due to severe abrasion. Edge chipping reveals the role of cryogenic cooling, causing the material to behave in a brittle manner with increased hardness. More tool-wear studies will be carried out in the next phase of this work to characterize the flank wear and its impact on the achievable surface quality and integrity.

## 4. Conclusions

The cryogenic drilling characteristics of AZ31-magnesium reinforced with hollow alumina microspheres were investigated through surface quality and integrity, machining forces, and burr formation. The following are the conclusions from this study:

1. Cryogenic drilling generated a 45% higher magnitude of thrust forces compared to dry and wet machining conditions while cutting AZ31-magnesium 15 vol% syntactic foam.
2. Thrust forces during cryogenic machining increased by 250% due to an increase in feed/tooth (0.05 mm/tooth to 0.6 mm/tooth) and by 87% with decreasing cutting speed (120 m/min to 40 m/min).
3. During cryogenic drilling, the higher the volume fraction of hollow alumina microspheres (15%) and the finer the average size of the microspheres (0.3 mm), the higher was the thrust forces by 45% and 36%, respectively.
4. Cryogenic machining (0.42 µm) produced a 75% improvement in surface roughness (Ra) values compared to wet machining (1.84 µm) with minimal subsurface machining-induced defects.
5. The surface roughness (Ra) deteriorated by approximately 125% with an increasing volume fraction of alumina microspheres (0.61 µm to 1.4 µm) under dry machining and cryogenic drilling (0.19 µm to 0.42 µm).
6. Burr height reduction of 53% was achieved with cryogenic machining (60 µm) compared to dry machining (130 µm) and wet machining using Almag^®^ Oil.
7. In summary, cryogenic hole drilling of AZ31 magnesium syntactic foams reinforced with hollow alumina ceramic microspheres results in good surface quality and integrity compared to dry machining and wet machining conditions.

## Figures and Tables

**Table 1:** Chemical composition of the hollow aluminum oxide.

Avg Bubble Size (mm)	Al_2_O_3_	Fe_2_O_3_	CaO	SiO_2_	Na_2_O	Bulk Density (Kg/m^3^)
0.3 mm, 0.6 mm	99.7	0.006	0.013	0.026	0.27	1700

**Table 2:** Properties of twist drills.

Property	TiAlN-PVD Coated	Uncoated
Flutes	3	3
Shank type	Straight	Straight
Drill diameter (mm)	5	5
Shank diameter (mm)	6	6
Grade	TiAlN Multilayer fine grain grade: KC7325	Solid carbide K10
Point angle	140°	140°
Helix angle	30°	30°
Flute length (mm)	20	35

**Table 3:** Experiment conditions.

Experiment Conditions		
Matrix	AZ31	Magnesium
Reinforcement	Hollow Alumina	Microsphere syntactic foam
Bubble volume fraction	(Vol %)	5%, 10%, 15%
Cutting speed	m/min	40–120
Feed per tooth	mm/tooth	0.05, 0.2, 0.4, 0.6
Hole depth	mm	5
Cutting insert	Kennametal™	TiAlN PVD coated K10 uncoated carbide
Cooling method	Cryogenic, Wet and Dry	
